# How central obesity influences intra-abdominal pressure: a prospective, observational study in cardiothoracic surgical patients

**DOI:** 10.1186/s13613-016-0195-8

**Published:** 2016-10-10

**Authors:** Marije Smit, Maureen J. M. Werner, Annemieke Oude Lansink-Hartgring, Willem Dieperink, Jan G. Zijlstra, Matijs van Meurs

**Affiliations:** Department of Critical Care (BA 49), University Medical Center Groningen, University of Groningen, PO Box 30001, 9700 RB Groningen, The Netherlands

**Keywords:** Intra-abdominal pressure, Central obesity, Waist/hip ratio, Waist circumference, Body mass index, Renal failure, Acute kidney injury

## Abstract

**Background:**

Intra-abdominal hypertension (IAH) is frequently present in critically ill patients and is an independent predictor for mortality. Better recognition of clinically important thresholds is necessary. Increased intra-abdominal pressure (IAP) is associated with renal dysfunction, and renal failure is one of the most consistently described organ dysfunctions associated with IAH. Obesity is also associated with kidney injury. The underlying mechanisms are not yet fully understood. Increased IAP may be a link in this association. The aim of this study was firstly to find the range in values of intra-abdominal pressure (IAP) in cardiothoracic surgery patients a secondly to investigate the relationship between central obesity, body mass index (BMI) and IAP and thirdly to investigate the relationship between IAP, inflammation and renal function in this population.

**Methods:**

Consecutive adult patients admitted to the cardiothoracic unit of the intensive care unit (ICU) after undergoing elective cardiothoracic surgery were included in this prospective, observational study. C-reactive protein (CRP) as a marker of inflammation and serum creatinine as a marker of renal function were measured pre- and postoperatively. Estimated glomerular filtration rates were calculated pre- and postoperatively. BMI was calculated. Waist circumference (WC), hip circumference (HC) and transvesical IAP were measured once directly after admission to the ICU postoperatively. Waist/hip ratio (WHR) was calculated (WC divided by HC). Three definitions of central obesity were used. Central obesity was defined according to WC, WHR or median WHR.

**Results:**

In total, 186 patients undergoing cardiothoracic surgery were included. Mean IAP was 9.1 mmHg (SD 4.4). IAP ≥ 12 mmHg was observed in 50 patients (26.9 %). IAP > 20 mmHg was measured in 4 patients (2.2 %). There was a positive correlation between IAP and BMI (*r* ^2^ = 0.05, *p* = 0.003). Correlations between IAP and WC (*r* ^2^ = 0.02, *p* = 0.054) and between IAP and WHR (*r* ^2^ = 0.01, *p* = 0.173) were not significant. There were no correlations between pre- or postoperative CRP and IAP (*r* ^2^ = 2.3 × 10^−4^, *p* = 0.839 and *r* ^2^ = 0.013, *p* = 0.117, respectively). In obese patients postoperative CRP was significantly higher than in non-obese patients (*p* = 0.034). There were no correlations between pre-operative serum creatinine and IAP (*r* ^2^ = 3.3 × 10^−5^, *p* = 0.938) or postoperative serum creatinine and IAP (*r* ^2^ = 0.003, *p* = 0.491).

**Conclusions:**

The range in IAP in patients undergoing cardiothoracic surgery was wide. There was a positive correlation between IAP and BMI. Correlations between IAP and indices for central obesity were not significant. In a multiple regression model BMI was a better predictor of IAP than WHR in this population. There were no correlations between pre- or postoperative CRP and IAP. Furthermore, this study did not find evidence for a relation between IAP and pre- and postoperative serum creatinine.

**Electronic supplementary material:**

The online version of this article (doi:10.1186/s13613-016-0195-8) contains supplementary material, which is available to authorized users.

## Background

Intra-abdominal hypertension (IAH) is frequently present in critically ill patients and is an independent predictor for mortality [1, 2]. Better understanding of the risks associated with IAH is necessary, as well as recognizing clinically important thresholds [3]. In order to recognize clinically important thresholds, we need to improve our understanding of the range in intra-abdominal pressure (IAP) values. Body mass index (BMI) is positively correlated with baseline IAP [4–9]. The consensus definitions published by the World Society of the Abdominal Compartment Syndrome (WSACS) state that normal IAP is approximately 5–7 mmHg in critically ill adults [10]. Baseline levels are higher at approximately 9–14 mmHg in morbidly obese patients [11]. These data were derived from several studies in a limited amount of patients. A possible explanation for higher pressures in the obese is that there could be a direct mass effect of the abdominal adipose tissue on the measurement of IAP [11]. Epidemiological studies have shown BMI as an index of general obesity, whereas waist circumference (WC) and waist/hip ratio (WHR) are indices of central obesity [12].

There are few studies describing the relationship between central obesity and IAP, and these studies have only been conducted in morbidly obese patients undergoing gastric bypass surgery. In a morbidly obese population, the IAP correlated with the sagittal abdominal diameter, an index of the degree of central obesity [13, 14]. WHR correlated with IAP in men but not in women [14].

In critically ill patients obesity is associated with acute kidney injury (AKI) [15]. Central obesity is associated with an unfavorable pattern of renal hemodynamic measures independent of BMI [16]. Multiple mechanisms may play a role, but these are not yet fully understood. Inflammation has been postulated to mediate at least part of the association between metabolic changes and chronic kidney disease [17]. The kidneys seem to be especially vulnerable to dysfunction induced by IAH [18, 19], and renal failure is one of the most consistently described organ dysfunctions associated with IAH [18, 20]. Increased IAP is correlated with renal dysfunction in advanced congestive heart failure, and IAP may be a link to explain why patients will eventually end up in dialysis [21]. Further data supporting this concept may be obtained by demonstrating the association of higher IAP values in obese patients in this study.

The aim of this study was firstly to find the range in values of IAP after cardiothoracic surgery. Treatment both in the operating room and in the intensive care unit (ICU) is strictly protocolized for cardiothoracic patients, although the indications for surgery may vary. Therefore, we measured IAP in patients who were admitted to the ICU after undergoing cardiothoracic surgery. The second aim was to investigate the relationship between central obesity, BMI and IAP in this population. We hypothesized that central obesity may be associated with elevated IAP and may be a better predictor of IAP than BMI. The third aim was to investigate the relationship between IAP, inflammation and renal function.

## Methods

Consecutive adult patients admitted to the cardiothoracic unit of the ICU after undergoing elective cardiothoracic surgery at a tertiary hospital from October 9, 2014, to March 31, 2015, were included in this prospective, observational study. Exclusion criteria were emergency surgery, chronic renal failure or dialysis, pregnancy, age <18 years and absence of a urinary bladder.

Waist and hip circumference and IAP were measured once directly after admission to the ICU postoperatively. BMI was calculated. C-reactive protein (CRP) as a marker of inflammation and serum creatinine as a marker of renal function were measured pre-operatively and on the first postoperative day. Estimated glomerular filtration rate (eGFR) was calculated pre- and postoperatively in each patient according to the CKD-EPI creatinine equation [22]. Delta GFR was calculated by subtracting pre-operative eGFR from postoperative eGFR.

Cardiothoracic surgery patients are routinely intubated and deeply sedated by the combination of intravenous opiates and continuous infusion of propofol upon postoperative admission to the ICU, at the time of IAP measurement. All patients were ventilated according to a lung protective strategy, including tidal volumes <7 ml/kg. Data were collected during the first postoperative admission only. Major abdominal symptoms were recorded during the first 30 postoperative days. Since IAP, CRP and serum creatinine measurements are all part of the standard care in this ICU, the Medical Ethics Board of the University Medical Center Groningen waived formal approval and consent (METc 2015/488).

### Anthropometric measurements

BMI as a measure of overall obesity was calculated by dividing body weight by height squared (kg/m^2^).

Waist circumference (WC) and hip circumference (HC) were measured postoperatively, after an overnight fast. WC was measured on bare skin, at the natural indentation between the 10th rib and iliac crest at the end of normal expiration to avoid influence of respiration phase on measurements. HC was measured at the region of the trochanter major. Values of WC and HC were expressed in whole centimeters, and waist/hip ratio (WHR) was calculated as WC divided by HC.

### Definitions

Normal weight was defined as BMI <25 kg/m^2^. Overweight was defined as BMI between 25 and 29.9 kg/m^2^, while obesity was defined as BMI ≥30 kg/m^2^ [23, 24].

The non-obese group was defined as both the normal weight and the overweight group.

Three definitions of central obesity were used:

1. A WC of ≥102 cm for males and ≥88 cm for females [24].
2. A WHR ≥0.90 for males and ≥0.85 for females [24].
3. A WHR > median in the study group [16].

The definition of IAH is a sustained IAP ≥ 12 [10]. Abdominal compartment syndrome (ACS) occurs when a sustained IAP > 20 is found when associated with new organ dysfunction or failure [10]. A major abdominal symptom was defined as a consultation by an abdominal surgeon and/or abdominal surgery within the first 30 postoperative days. Acute kidney injury (AKI) was defined by the RIFLE criteria using pre-operative and day 1 postoperative serum creatinine and eGFR levels only [25]. Comorbidity was recorded using APACHE IV definitions.

### Intra-abdominal pressure

Transvesical IAP was measured according to a standard protocol using 25 ml of sterile saline as priming volume with the symphysis pubis as the zero reference point. Patients were in the supine position during IAP measurement.

### Statistical analysis

IBM SPSS Statistics 22 was used for statistical analysis. Descriptive statistics were used. Dichotomous data were presented by proportions and continuous data by means with standard deviations. Independent Student’s t tests were used to calculate the differences between the male and female groups and between the non-obese and obese groups for continuous data as recorded in Table 1. Chi-square tests were used to calculate differences between the groups for the dichotomous data in Table 1. Mann−Whitney *U* tests were used to analyze differences in mechanical ventilation duration and length of ICU between groups. Paired samples *t* tests were performed to calculate the differences between pre- and postoperative CRP, serum creatinine and between pre- and postoperative estimated GFR.

**Table 1:** Patient characteristics

	Total number of patients	Male	Female	*p* value	Non-obese	Obese	*p* value
Total number of patients	186	138 (74 %)	48	N/A	148	38	N/A
Male	138	N/A	N/A	N/A	111	27	N/A
Female	48	N/A	N/A	N/A	37	11	N/A
Age (years), mean (SD)	64.2 (11.6)	64.2 (10.8)	64.1 (13.5)	0.962	64.4 (12.1)	63.1 (9.3)	0.535
Height (cm), mean (SD)	175.2 (9.9)	179.0 (7.4)	164.3 (7.9)	*<0.01*	175.6 (10.0)	173.5 (9.6)	0.225
Weight (kg), mean (SD)	82.4 (14.1)	86.1 (11.9)	71.9 (14.8)	*<0.01*	78.6 (12.3)	97.4 (10.3)	*<0.01*
BMI (kg/m^2^), mean (SD)	26.8 (3.8)	26.8 (3.2)	26.6 (5.2)	0.793	25.4 (2.5)	32.4 (2.6)	*<0.01*
WC (cm), mean (SD)	101.9 (10.8)	102.6 (9.7)	99.7 (13.3)	0.168	98.8 (9.4)	113.8 (6.7)	*<0.01*
HC (cm), mean (SD)	106.1 (8.7)	105.3 (7.4)	108.2 (11.5)	0.108	103.5 (6.9)	116.2 (7.6)	*<0.01*
WHR, mean (SD)	0.96 (0.07)	0.97 (0.06)	0.92 (0.07)	*<0.01*	0.95 (0.07)	0.98 (0.06)	*0.025*
IAP (mmHg), mean (SD)	9.1 (4.4)	9.2 (4.0)	8.6 (5.2)	0.398	8.7 (4.2)	10.4 (4.7)	*0.031*
IAP ≥ 12	50	40	10	0.086	35	15	0.667
Pre-operative creatinine (umol/l), mean (SD)	86.0 (19.7)	89.3 (19.5)	76.4 (17.3)	*<0.01*	85.5 (19.4)	87.7 (21.1)	0.546
Pre-operative CRP (mg/l), mean (SD)	7.3 (13.2)	7.2 (13.3)	7.6 (13.3)	0.884	6.7 (12.8)	9.7 (14.7)	0.211
Pre-operative eGFR (ml/min/1.73 m^2^)	78.2 (17.2)	79.6 (16.8)	73.9 (17.8)	*0.046*	78.5 (17.0)	77.0 (17.9)	0.627
Postoperative creatinine (umol/l), mean (SD)	74.1 (21.8)	77.5 (21.5)	64.4 (19.9)	*<0.01*	73.2 (22.0)	77.9 (21.0)	0.230
Postoperative CRP (mg/l), mean (SD)	49.5 (30.1)	52.2 (31.6)	41.9 (23.8)	*0.040*	46.5 (26.6)	61.1 (39.0)	*0.034*
Postoperative eGFR (ml/min/1.73 m^2^)	88.6 (18.6)	89.2 (18.3)	86.7 (19.8)	0.426	89.4 (18.8)	85.6 (17.8)	0.265
Delta eGFR, mean (SD) (ml/min/1.73 m^2^)	10.4 (10.2)	9.6 (9.7)	12.8 (11.4)	0.059	10.9 (9.9)	8.6 (11.4)	0.223
Comorbidity Chronic diagnosis at admission ICU							
COPD	17	13	4	N/A	14	3	N/A
Chronic cardiovascular insufficiency	10	8	2	N/A	8	2	N/A
Immunological insufficiency	8	6	2	N/A	4	4	N/A
Metastasized neoplasm	3	2	1	N/A	2	1	N/A
Respiratory insufficiency	1	1	0	N/A	1	0	N/A
Hemotological malignancy	1	1	0	N/A	1	0	N/A
Diagnosis at admission ICU							
Cardiovascular resuscitation	1	1	0	N/A	1	0	N/A
Dysrhythmia	2	1	1	N/A	2	0	N/A
Mechanical ventilation at admission	186	138	48	N/A	148	38	N/A
Diabetes	21	12	9	0.103	11	10	*0.003*
Myocardial infarction	40	34	6	0.119	32	8	1.0
Diagnosis 24 h after ICU admission							
Acute kidney injury	1	1	0	N/A	1	0	N/A
Confirmed infection	1	1	0	N/A	1	0	N/A
Vasoactive medication in first 24 h	164	119	45	0.259	132	32	0.571
Apache IV score, mean (SD) N = 184	42.8 (13.8)	41.8 (15.0)	43.7 (12.5)	0.435	42.0 (15.3)	43.4 (10.1)	0.521
Euro score, mean (SD)	7.1 (7.6)	6.4 (7.4)	8.9 (7.9)	*0.050*	7.6 (8.1)	5.2 (4.7)	*0.024*
Surgery							
CABG	97	80	17	N/A	74	23	N/A
CABG + valve	16	9	7	N/A	13	3	N/A
Valve	58	40	18	N/A	49	9	N/A
Aneurysm—thoracic aortic repair	9	5	4	N/A	8	1	N/A
Other^a^	6	4	2	N/A	4	2	N/A
Operating time (min), mean (SD)							
*N = 179*	245.9 (88.1)	247.7 (92.1)	240.7 (76.0)	0.642	240.9 (80.1)	265.1 (113.1)	0.138
Perfusion time (min), mean (SD)							
*N = 117*	149.8 (71.7)	151.4 (78.0)	146.0 (55.9)	0.707	143.7 (64.5)	177.6 (95.4)	*0.049*
Aorta occlusion time (min), mean (SD)							
*N = 101*	104.9 (49.0)	106.4 (53.3)	101.7 (38.7)	0.656	102.2 (45.7)	116.5 (61.4)	0.255
ICU							
Mechanical ventilation duration in hours, median (range)	8.0 (187)	7.0 (187)	8.0 (91)	0.402	8.0 (187)	8.0 (85)	0.335
Reintubation within 72 h after detubation	0	0	0	N/A	0	0	N/A
Length of stay ICU in days, median (range)	0.91 (19.4)	0.91 (19.4)	0.92 (6.1)	0.588	0.91 (19.4)	0.94 (5.2)	0.060

*p* < 0.05 is significant

*N/A* not applicable

^a^Includes pericardiectomy, congenital defect repair and ablation

One-way between-group ANOVA was used to calculate the difference in IAP in normal weight, overweight and obese patients. Levels of effect size eta squared were calculated and interpreted as follows: eta squared 0.01 small effect, 0.06 medium effect, 0.14 large effect [26]. Pearson’s bivariate correlations were used to investigate the relationship between central obesity, BMI, IAP, CRP, serum creatinine and eGFR. Levels of correlation were interpreted as follows:

- No correlation *r* < 0.10 (*r* ^2^ < 0.01)
- Small correlation *r* = 0.10 to 0.29 (*r* ^2^ = 0.01 to 0.08)
- Medium correlation *r* = 0.30 to 0.49 (*r* ^2^ = 0.09 to 0.24)
- Large correlation *r* = 0.50 to 1.0 (*r* ^2^ = 0.25 to 1) [26]

A standard multiple regression model was used to investigate whether BMI or WHR can better predict IAP and how much variance in IAP can be explained by values on these 2 scales.

## Results

**Fig. 1:**
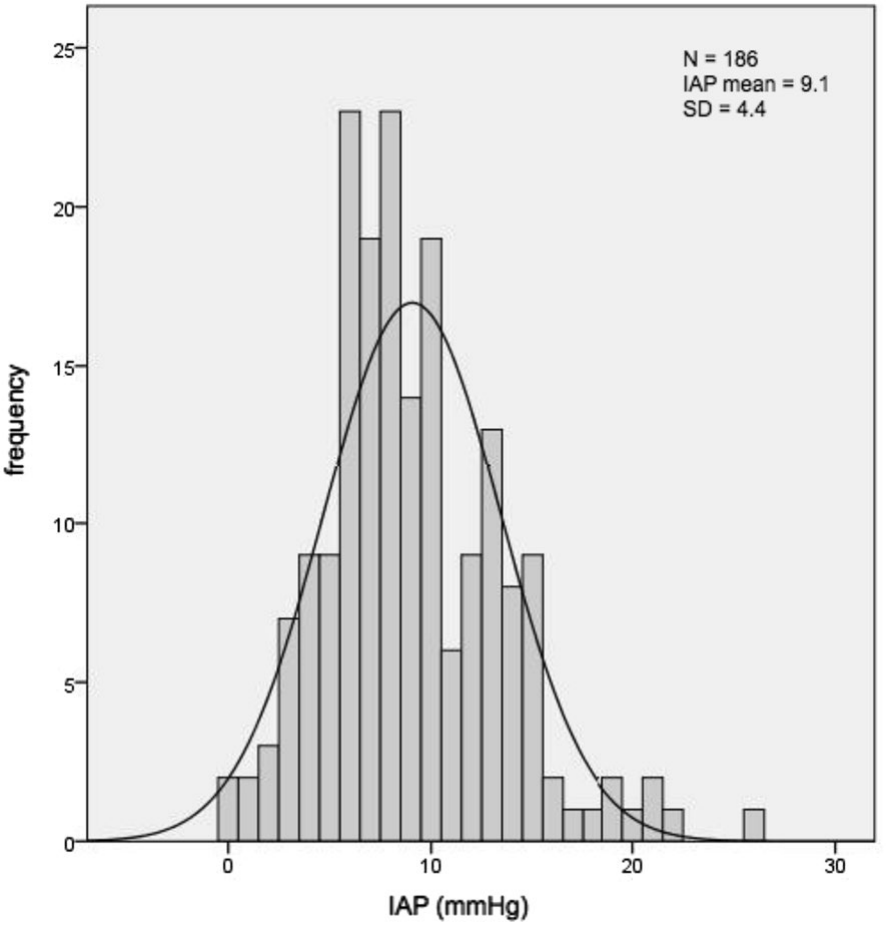
Histogram showing IAP distribution

In total, 186 patients were included, 138 males and 48 females (Table 1). Data in Table 1 include comparisons by gender and by the absence or presence of obesity. All patients had elective surgery, which was most often (52 %) a coronary artery bypass grafting (CABG). Mean operating time was 245.9 min (SD 88.1 min). In 117 patients (63 %) extracorporeal circulation was used with a mean perfusion time of 149.8 min (SD 71.7 min). All patients were mechanically ventilated upon admission to the ICU. Mechanical ventilation was continued for a median of 8 h (range 3–190 h). Most patients (88 %) required at least one type of vasoactive medication during their stay in the ICU. The median length of stay in the ICU was 0.91 days (range 0.4–19.7 days). Missing data were recorded in Table 1, when applicable. Figure 1 shows IAP distribution in this population. IAP values ranged from 0 to 26 mmHg. Mean IAP was 9.1 mmHg (SD 4.4). IAP ≥ 12 mmHg was observed in 50 patients (26.9 %); 24 of these patients were overweight and 15 patients were obese. IAP > 20 mmHg was measured in 4 patients (2.2 %); 2 of these patients were obese, and 1 was overweight. Three patients (1.6 %) developed major abdominal symptoms during the first 30 days postoperatively. These 3 patients all had an IAP < 12 mmHg. One patient had a perforation of a Zenker’s diverticulum due to perioperative placement of a gastric tube, 1 patient had coprostasis, and 1 patient had abdominal pain due to pneumonia. Management was conservative in all cases.

**Fig. 2:**
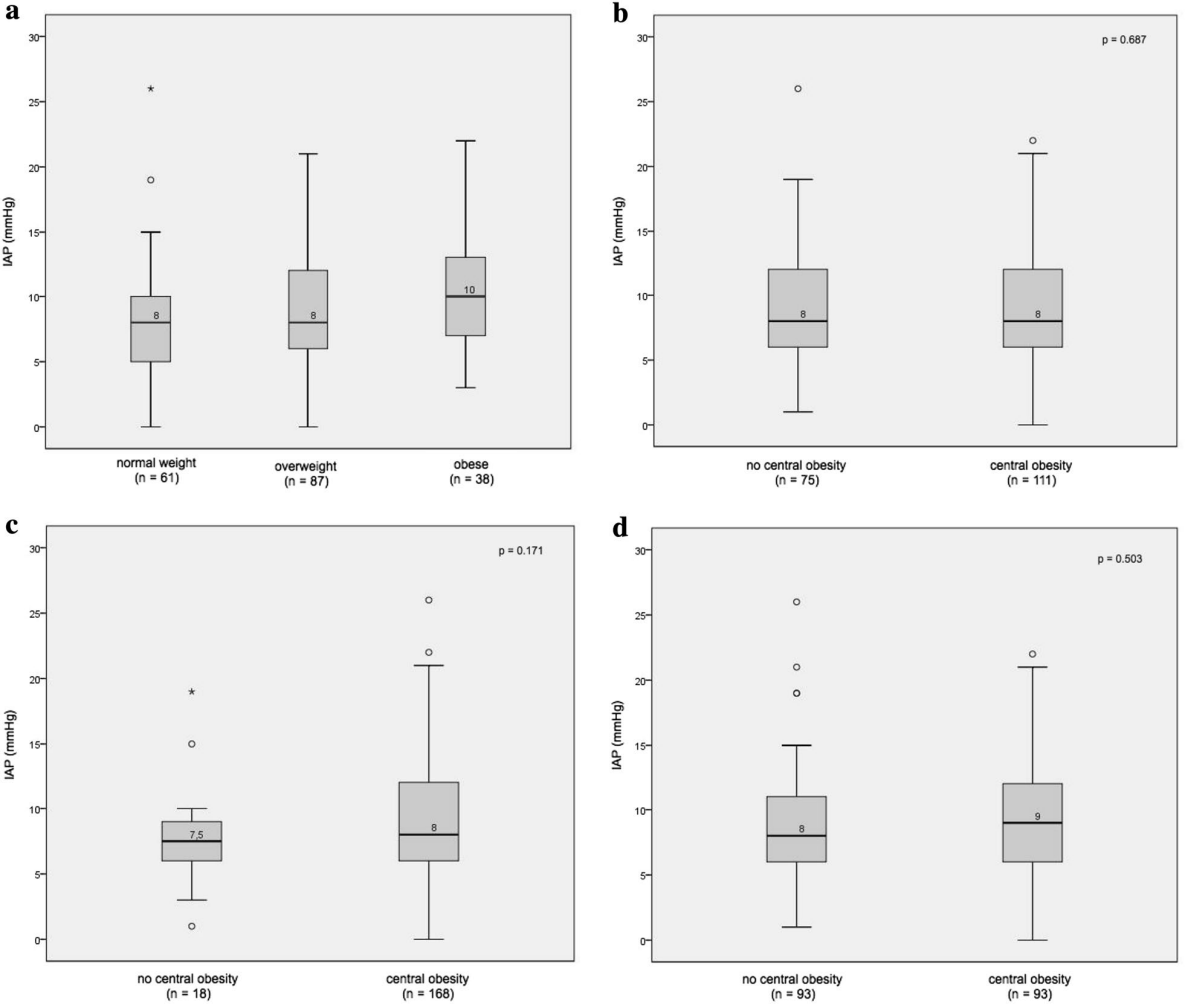
IAP distribution dependent on definition. **a** IAP distribution according to BMI. **b** IAP distribution according to WC. **c** IAP distribution according to WHR. **d** IAP distribution according to median WHR

Table 1 shows that IAP is significantly higher in the obese group when compared with the non-obese group. Mean IAP was 10.4 mmHg (SD 4.7) in the obese group and 8.7 mmHg (SD 4.2 mmHg) in the non-obese group (*p* = 0.031). Figure 2 shows IAP distribution dependent on definition. Firstly IAP distributions versus BMI are shown in Fig. 2a. Mean BMI was 26.8 kg/m^2^ (SD 3.8). In total, 87 patients were overweight and 38 patients were obese. Mean IAP was different between different weight groups (*p* = 0.029). In the normal weight group mean IAP was 8.1 mmHg (SD 4.6), in the overweight group 9.2 mmHg (SD 3.9) and in the obese group 10.4 mmHg (SD 4.7). Firstly, there was a significant difference between the obese group and the other two groups (*p* = 0.023) and secondly there was a significant difference when the obese group was compared to the normal weight group (*p* = 0.008). The difference in mean IAP between the groups was small. The effect size was small (eta squared = 0.04).

In Fig. 2b–d IAP distribution is shown when central obesity is absent or present according to the definitions 1–3 of central obesity. The number of patients with central obesity varied according to which definition was used. If central obesity was defined according to WC (definition 1), there were 111 patients (59.7 %) with central obesity in this population. Mean IAP was 8.9 (SD 4.4) when central obesity was absent and 9.2 (SD 4.3) when central obesity was present (*p* = 0.687). Both groups had a median IAP of 8.0 mmHg.

If central obesity was defined according to WHR (definition 2), there were 168 patients (90.3 %) with central obesity in this population. Using this definition, mean IAP was 7.7 mmHg (SD 4.2) when central obesity was absent and 9.2 mmHg (SD 4.4) when central obesity was present (*p* = 0.171).

If central obesity was defined according to median WHR (=0.96) (definition 3), there were 93 patients (50 %) with central obesity in this population. Using this definition, mean IAP was 8.9 mmHg (SD 4.4) when central obesity was absent and 9.3 mmHg (SD 4.3) when central obesity was present (*p* = 0.503). Although there was a trend toward a higher mean IAP in central obesity, regardless of which definition was used, this was not statistically significant.

**Fig. 3:**
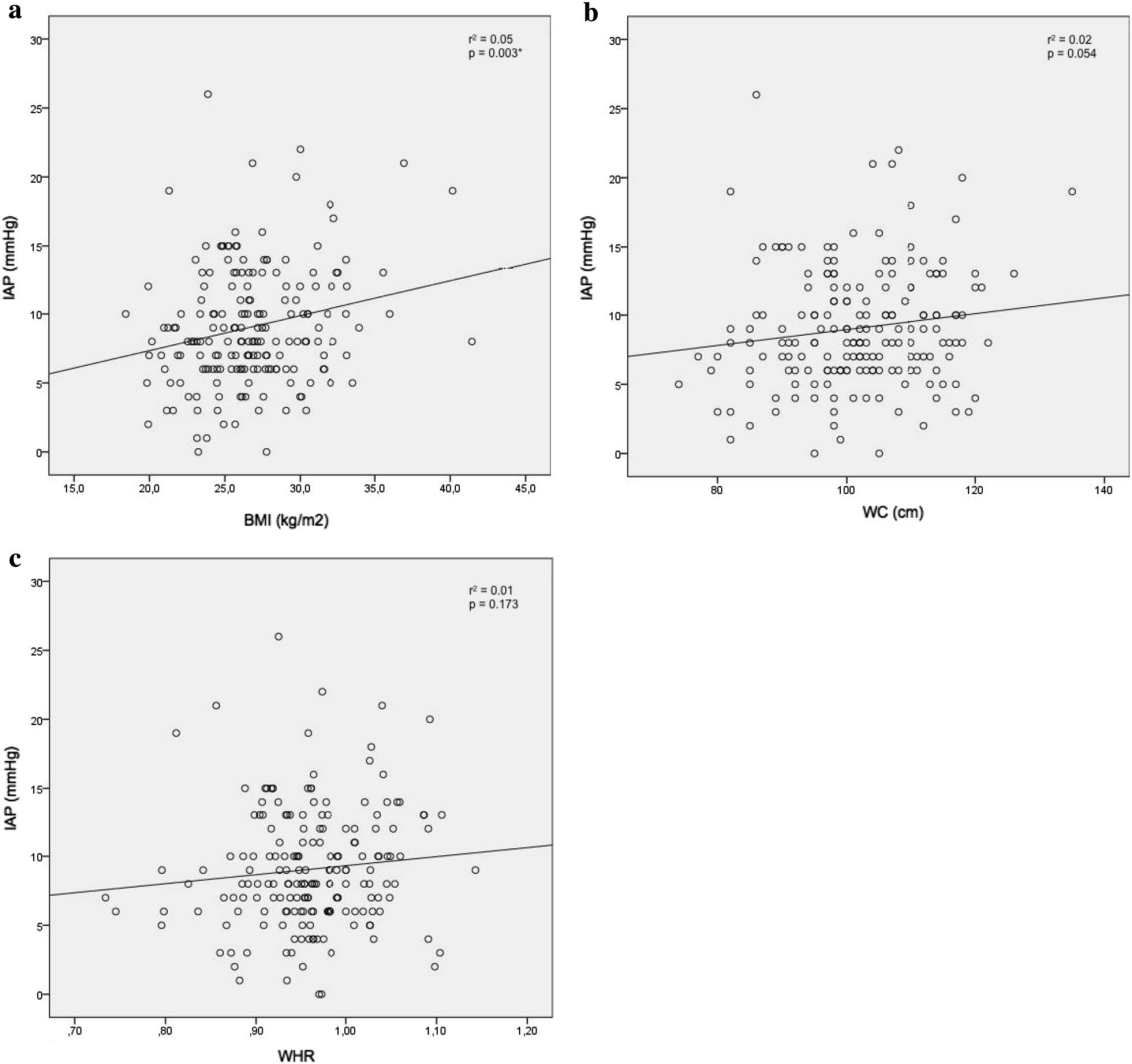
**a** Correlation between IAP and BMI. **b** Correlation between IAP and WC. **c** Correlation between IAP and WHR

There was a positive correlation between IAP and BMI (*r* ^2^ = 0.05, *p* = 0.003). Correlations between IAP and WC (*r* ^2^ = 0.02, *p* = 0.054) and between IAP and WHR (*r* ^2^ = 0.01, *p* = 0.173) were not significant (Fig. 3). A multiple regression model where BMI and WHR were analyzed as predictors of IAP showed that BMI and WHR together explained 5 % of the variance in IAP (*p* = 0.009). BMI made a significant unique contribution to IAP (Beta 0.208, *p* = 0.006) which was greater than the unique contribution of WHR (Beta 0.043, *p* = 0.569). Hence, BMI is a better predictor of IAP than WHR.

Additional file 1: Figure S1 shows IAP distribution in non-obese and obese patients.

There was no correlation between IAP and operating time (*r* ^2^ = 0.006, *p* = 0.320). Correlations between IAP and perfusion time (*r* ^2^ = 0.01, *p* = 0.268) and between IAP and aorta occlusion time (*r* ^2^ = 0.01, *p* = 0.278) were not significant (Additional file 2: Figure S2). Mean IAP in patients with extracorporeal perfusion was 9.0 mmHg (SD 4.3) and without extracorporeal perfusion 9.2 mmHg (SD 4.4) (*p* = 0.741).

Mean CRP was 7.3 mg/l (SD 13.2) pre-operatively. It increased to a mean of 49.5 mg/L (SD 30.1, *p* < 0.05) on the first postoperative day. There was no correlation between pre- or postoperative CRP and IAP (pre-operative *r* ^2^ = 2.3 × 10^−4^, *p* = 0.839; postoperative *r* ^2^ = 0.013, *p* = 0.117) (Table 2). Postoperative CRP was correlated with BMI, WC and WHR (Table 2), and in obese patients postoperative CRP was significantly higher than in non-obese patients (*p* = 0.034) (Table 1).

**Table 2:** Correlations of pre- and postoperative CRP, serum creatinine and eGFR and IAP, BMI, WC and WHR

	IAP *r* ^2^	*p*	BMI *r* ^2^	*p*	WC *r* ^2^	*p*	WHR *r* ^2^	*p*
Pre-operative CRP	2.3 × 10^−4^	0.839	0.018	0.071	0.004	0.418	0.002	0.582
Postoperative CRP	0.013	0.117	0.052	*0.002*	0.034	*0.012*	0.055	*0.001*
Pre-operative serum creatinine	3.3 × 10^−5^	0.938	0.009	0.205	0.009	0.193	0.024	*0.033*
Postoperative serum creatinine	0.003	0.491	0.025	*0.030*	0.025	*0.031*	0.038	*0.008*
Pre-operative eGFR	4.3 × 10^−4^	0.780	0.006	0.275	0.015	0.097	0.003	0.435
Postoperative eGFR	5.4 × 10^−4^	0.753	0.019	0.062	0.035	*0.011*	0.015	0.092

*p* < 0.05 is significant

Mean serum creatinine pre-operatively was 86.0 umol/l (SD 19.7) and decreased postoperatively to 74.1 umol/l (SD 21.8, *p* < 0.05).

Mean eGFR pre-operatively was 78.2 (SD 17.2) and increased postoperatively to 88.6 ml/min/1.73 m^2^ (SD 18.6, *p* < 0.05). There was no correlation between pre-operative serum creatinine and IAP (*r* ^2^ = 3.3 × 10^−5^ (*p* = 0.938)) or postoperative serum creatinine and IAP (*r* ^2^ = 0.003 (*p* = 0.491)) (Table 2). Correlations between pre- and postoperative eGFR and IAP were *r* ^2^ = 4.35 × 10^−4^ (*p* = 0.780) and *r* ^2^ = 5.4 × 10^−4^ (*p* = 0.753), respectively. There were small correlations between postoperative serum creatinine and BMI, WC and WHR (Table 2). One patient developed acute kidney injury. According to the RIFLE criteria this patient is included in the risk category, with an increase in serum creatinine from 68 to 121 μmol/l and a 44 % decrease in eGFR. This male patient had a BMI of 29.4 kg/m^2^ and an IAP of 5 mmHg.

A subgroup analysis shows that in males with central obesity, defined by WHR, IAP was significantly higher than in males without central obesity (Additional file 3: Figure S3). Additional file 4: Figure S4 shows a flowchart of this study

## Discussion

This study shows a wide range in IAP in patients undergoing elective cardiothoracic surgery, between 0 and 26 mmHg. A single IAP measurement was elevated ≥12 mmHg in more than 26 % of patients. Furthermore, an IAP > 20 mmHg was measured in 4 patients. We found a correlation between BMI and IAP. We did not find that other definitions of body shape correlated better with IAP than BMI. We did not find a correlation between CRP or serum creatinine and IAP. However, postoperative CRP and postoperative serum creatinine are correlated with BMI, WC and WHR.

The large variation in IAP shows that a single measurement of IAP should be interpreted with caution. Ultimately, the clinical context gives meaning to an increased IAP and repeated measurements should be performed before a diagnosis of IAH or ACS can be made.

Most patients were either overweight or obese. The high proportion of obesity in this cardiothoracic surgery population is not surprising, considering obesity is a risk factor for cardiovascular conditions. Mean IAP in the obese group was significantly higher than in the non-obese group. However, despite reaching statistical significance, the actual difference in mean scores between the groups was small. Furthermore, BMI is a better predictor of IAP than WHR, but BMI and WHR together explained only 5 % of the variance in IAP. This means that in an obese patient with clinical symptoms of ACS an increased IAP should never be attributed to obesity only. There is no evidence that an obesity-related elevation in IAP is not a true ACS, but only a direct mass effect of the visceral obesity [13].

At least 50 % of patients in this study have central obesity. The exact number of patients with central obesity varies according to which definition is used. Although there was a trend toward a higher mean IAP in central obesity according to these definitions, this was not statistically significant. Even though there seemed to be a positive correlation between IAP and WC and between IAP and WHR, this was not statistically significant. Hence, this study does not match with findings from previous studies in the morbidly obese [13, 14]. We believe that this study may have been underpowered to show these relations. We did not find a correlation between IAP and inflammation or between IAP and renal function in this population. Future studies should consider using more sensitive biomarkers for determination of both systemic inflammation and renal function and take into account that larger datasets may be required to find relations.

We did find a correlation between postoperative CRP and postoperative serum creatinine and BMI, WC and WHR. This matches with findings in a larger group of otherwise healthy persons, where the correlation between renal function and body shape was also shown [16].

A subgroup analysis shows that in males with central obesity, defined by WHR, IAP was significantly higher than in males without central obesity. This difference was not found in females. This matches with findings in the morbidly obese, where WHR was correlated with transvesical IAP in men but not in women [14]. A possible explanation may be the difference in abdominal compliance between males and females. In patients with a decreased abdominal compliance, the same change in intra-abdominal volume will result in a greater change in IAP. Central obesity usually results in increased visceral fat and a sphere-like baseline shape of the abdominal cavity with poor stretching capacity, whereas in peripheral obesity the internal abdominal diameter is shaped as an ellipse and has a huge stretching capacity (and thus higher abdominal compliance) [27]. Factors associated with decreased abdominal compliance include male gender and (central) obesity. Factors associated with increased abdominal compliance include female gender, peripheral obesity, previous pregnancy and previous abdominal surgery [28].

We assumed that the patients in this study did not have an increased risk for development of IAH or ACS. However, there is one study in 25 patients which concludes that the CABG procedure with extracorporeal circulation may result in increased intra-abdominal pressure due to the invoked inflammatory response by the extracorporeal circulation [29]. Operating time, perfusion time and aorta occlusion time were not correlated with IAP in the present study. Furthermore, there was no difference in mean IAP in patients with and without extracorporeal circulation. Therefore, the findings in our study do not corroborate Dabrowski’s conclusions.

These results raise the question whether the elevations in IAP measured in this study are pathological. Since IAP was measured only once, a diagnosis of IAH or ACS could by definition not be made. Furthermore, only 3 patients developed abdominal symptoms during their stay in the ICU; none of these patients had an IAP ≥ 12 mmHg upon admission. However, higher IAP values were found in obese patients and we have to consider that the IAP in obese patients is chronically increased. Even slight elevations in IAP are associated with increased systemic inflammation, and signs of acute kidney injury [19] and weight excess and/or central body fat distribution are associated with increased long-term renal risk [30]. Obesity is associated with acute kidney injury in critically ill patients [15], and this study shows a correlation between postoperative serum creatinine and BMI, WC and WHR. IAP is probably only one of many contributing factors to renal function, and it will be hard to dissect consequences of a slight chronic increase in IAP from other factors related to renal function loss [31]. In contrast to acute models of ACS, there are no models of small long-term increases in IAP.

Since IAP > 12 independently predicts organ failure and mortality in a mixed population of critically ill patients [1, 2], perhaps we should monitor IAP more closely in overweight and obese patients when they are critically ill, in order to avoid any further increase in IAP.

### Limitations of this study

This study was performed in a selected population of elective cardiothoracic surgery patients. Moreover, other anthropomorphic parameters like sagittal abdominal diameter were not measured in this study.

IAP was measured only once per patient; therefore, a diagnosis of IAH (sustained or repeated IAP ≥ 12 mmHg) or ACS (sustained or repeated IAP > 20 with new organ failure) could not be made. Furthermore, IAP was measured postoperatively and this measurement could have been influenced by perioperative fluid management.

CRP was measured as a marker of inflammation, and serum creatinine was measured as a marker of renal function; however, these markers lack specificity and sensitivity to determine subtle differences in inflammation and renal function. This study may have been underpowered to show a relation between body shape and IAP, CRP and serum creatinine. Sensitive AKI biomarkers, such as neutrophil gelatinase-associated lipocalin (NGAL) and cystatin C, may reveal the relation between IAP and renal function in future studies with larger datasets.

## Conclusions

The range in IAP in patients undergoing cardiothoracic surgery was wide. There was a positive correlation between IAP and BMI. Correlations between IAP and indices for central obesity were not significant. In a multiple regression model BMI was a better predictor of IAP than WHR in this population. There were no correlations between pre- and postoperative CRP and IAP. Furthermore, this study did not find evidence for a relation between IAP and pre- and postoperative serum creatinine.

## Additional files

10.1186/s13613-016-0195-8 **A** IAP distribution in non-obese patients. **B**. IAP distribution in obese patients.

10.1186/s13613-016-0195-8 **A** Correlation between IAP and operating time. **B**. Correlation between IAP and perfusion time. **C** Correlation between IAP and aorta occlusion time.

10.1186/s13613-016-0195-8 **A** IAP distribution according to WC in males. **B**. IAP distribution according to WHR in males. **C** IAP distribution according to WC in females. **D**. IAP distribution according to WHR in females.

10.1186/s13613-016-0195-8 Flow chart.

## Abbreviations

ACS: abdominal compartment syndrome
IAH: intra-abdominal hypertension
IAP: intra-abdominal pressure
BMI: body mass index
CABG: coronary artery bypass grafting
WHR: waist/hip ratio
WC: waist circumference
ICU: intensive care unit
CRP: C-reactive protein
WSACS: World Society of Abdominal Compartment Syndrome
NGAL: neutrophil gelatinase-associated lipocalin
AKI: acute kidney injury
eGFR: estimated glomerular filtration rate
N/A: not applicable
