# Supplementary figures and images for: How central obesity influences intra-abdominal pressure: a prospective, observational study in cardiothoracic surgical patients
Source: Ann Intensive Care. 2016 Oct 10;6:99. doi: 10.1186/s13613-016-0195-8 (PMC5056912; doi:10.1186/s13613-016-0195-8)

**Figure S1A. IAP distribution in non-obese patients**

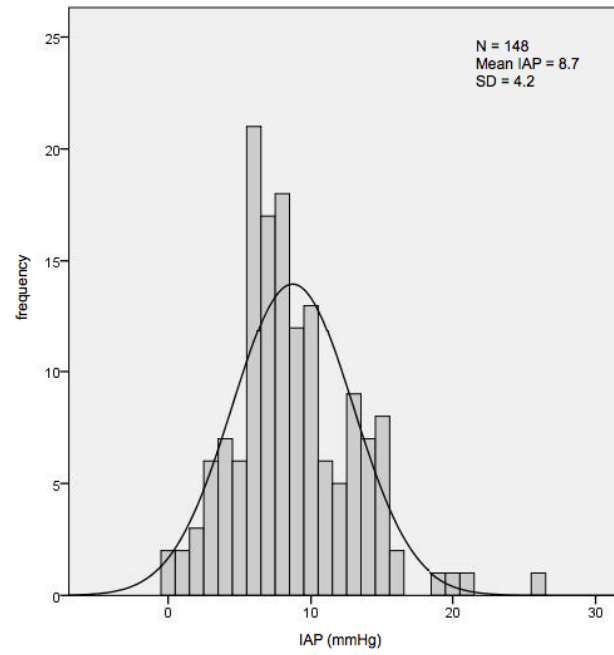

**Figure S1B. IAP distribution in obese patients**

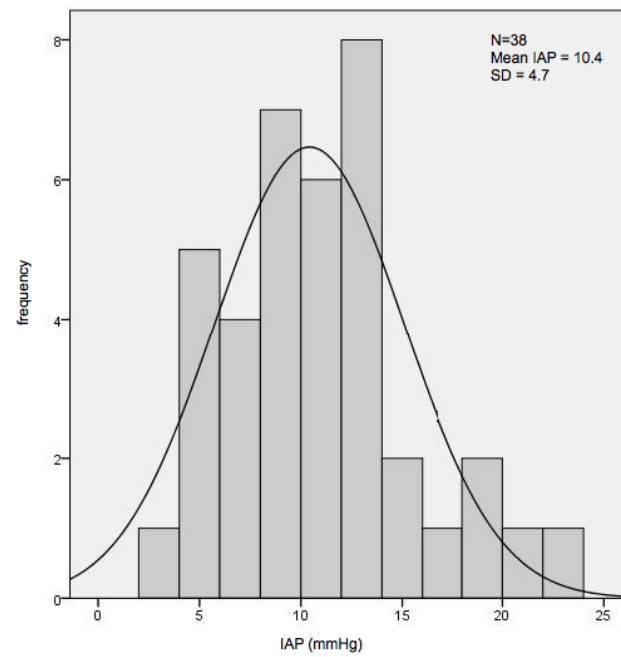

Supplement: Supplementary file 1 — 10.1186/s13613-016-0195-8 A IAP distribution in non-obese patients. B. IAP distribution in obese patients. [file 13613_2016_195_MOESM1_ESM.pdf]

Figure S3A. WC - Males

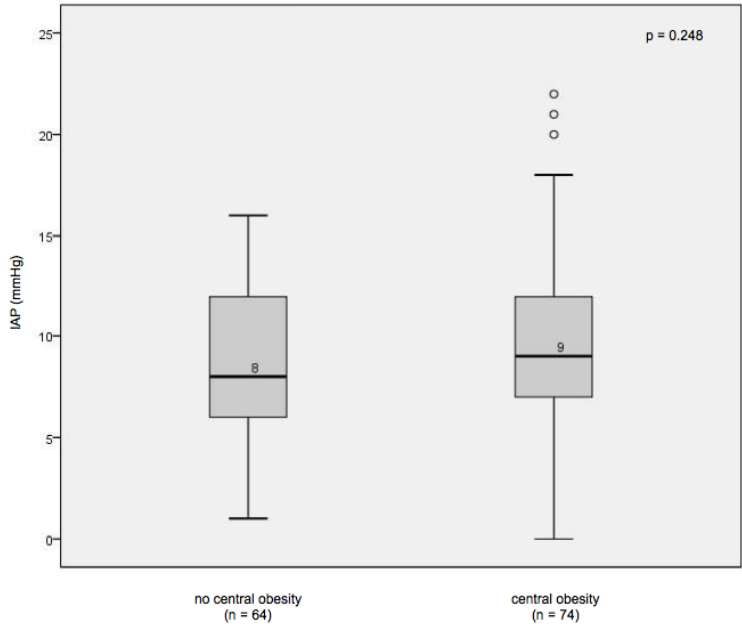

Figure S3B. WHR - Males

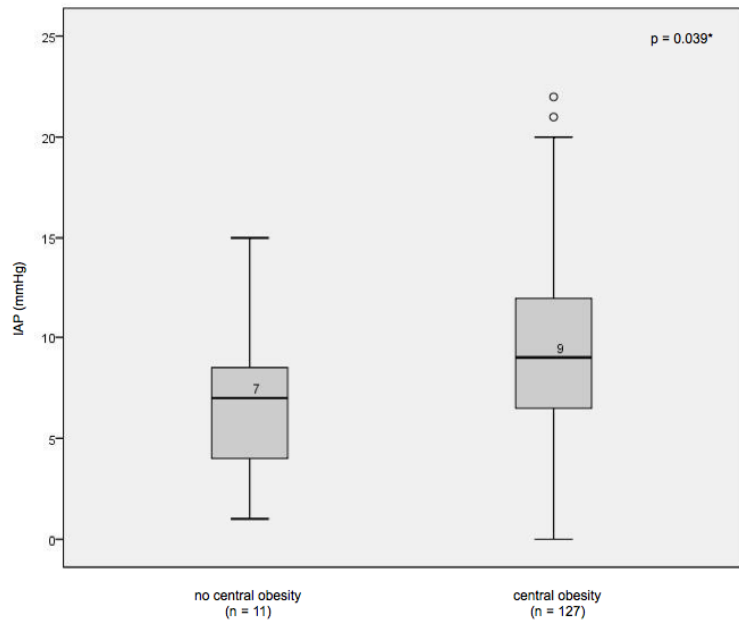

Figure S3C. WC - Females

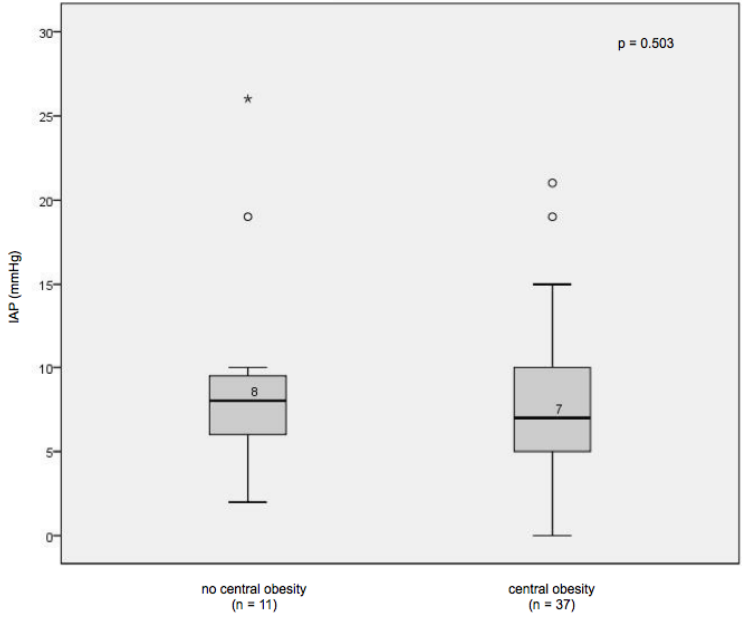

Figure S3D. WHR - Females

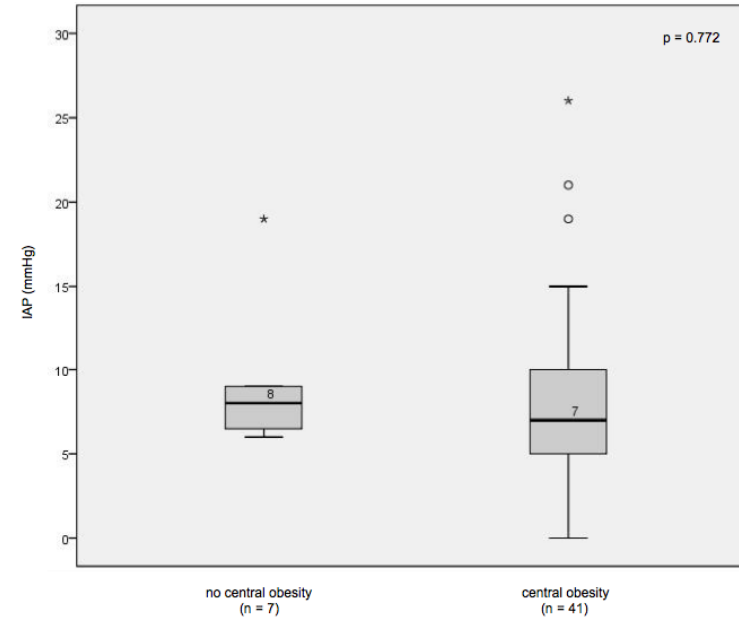

Supplement: Supplementary file 3 — 10.1186/s13613-016-0195-8 A IAP distribution according to WC in males. B. IAP distribution according to WHR in males. C IAP distribution according to WC in females. D. IAP distribution according to WHR in females. [file 13613_2016_195_MOESM3_ESM.pdf]

**Figure S4. Flow chart**

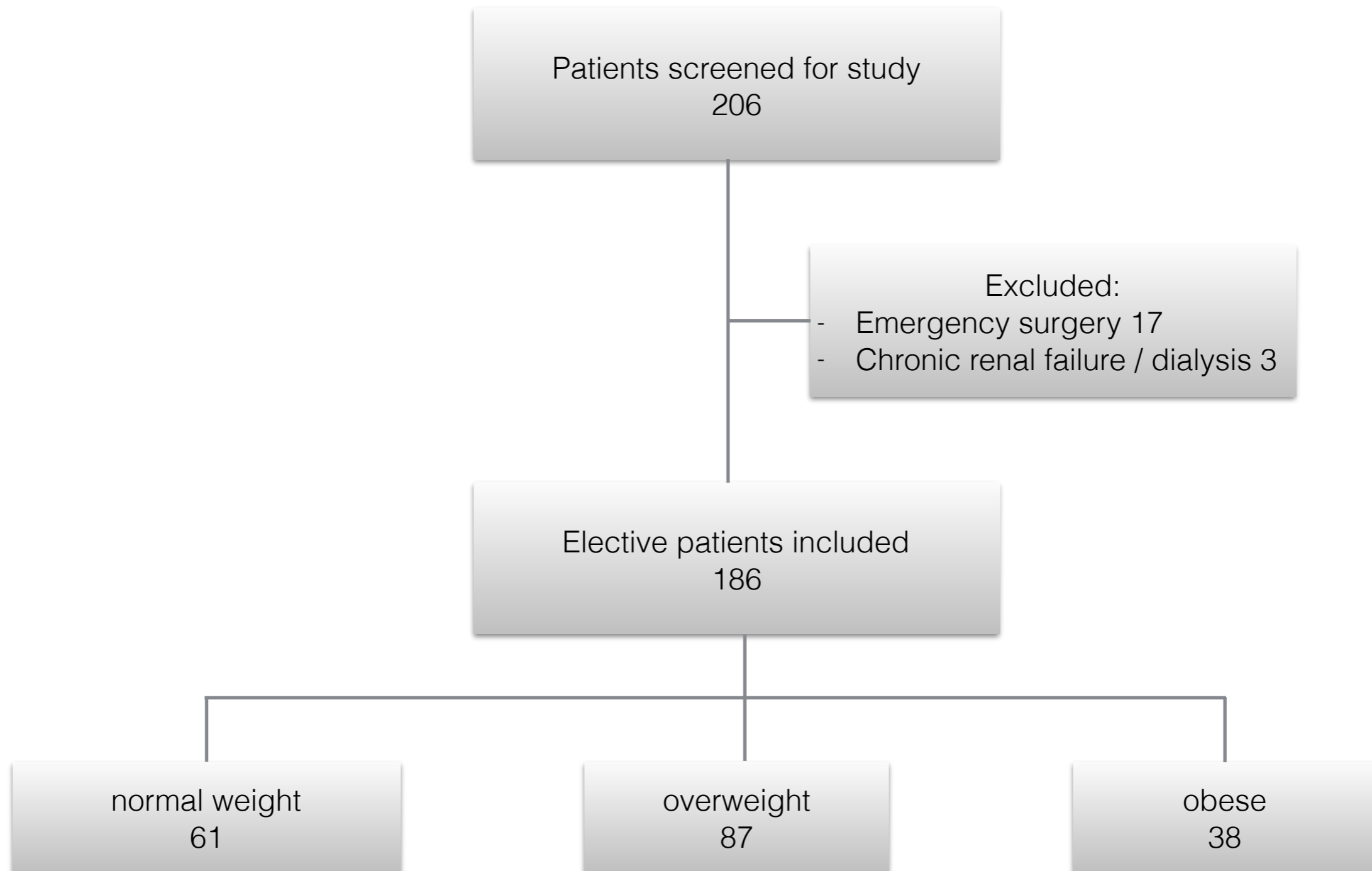

Supplement: Supplementary file 4 — 10.1186/s13613-016-0195-8 Flow chart. [file 13613_2016_195_MOESM4_ESM.pdf]
