# Supplementary material for: How central obesity influences intra-abdominal pressure: a prospective, observational study in cardiothoracic surgical patients
Source: Ann Intensive Care. 2016 Oct 10;6:99. doi: 10.1186/s13613-016-0195-8 (PMC5056912; doi:10.1186/s13613-016-0195-8)

Figure S2A. Correlation between IAP and operating time

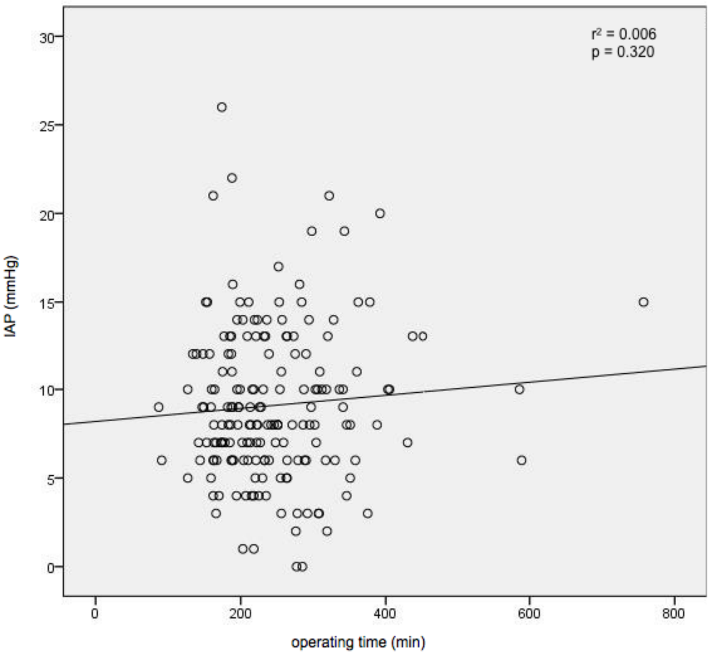

Figure S2B. Correlation between IAP and perfusion time

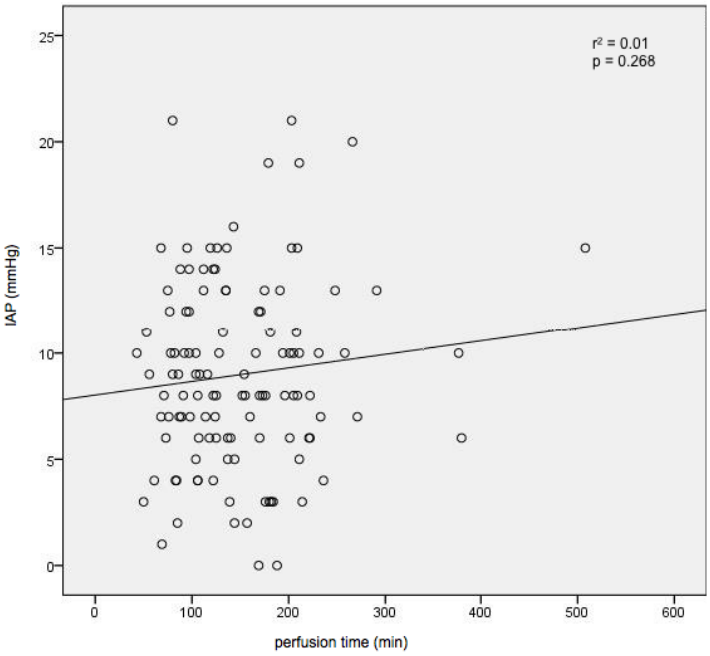

Figure S2C. Correlation between IAP and occlusion time

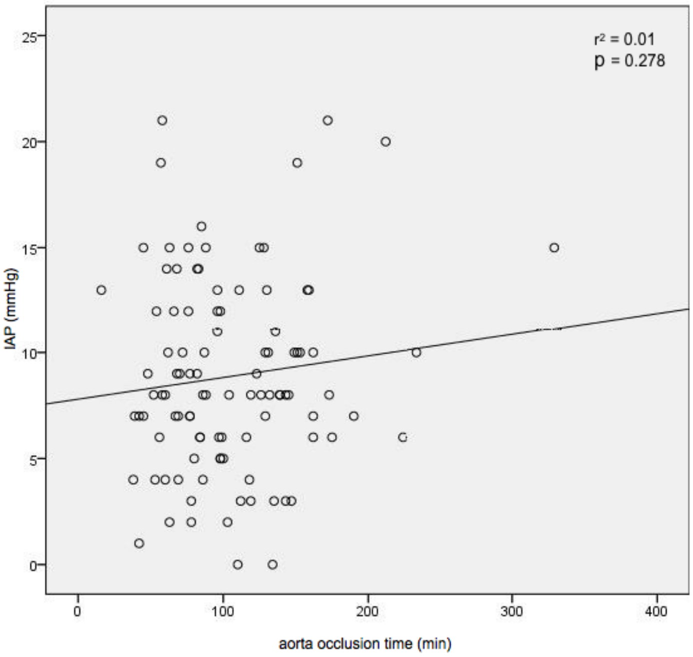

Supplement: Supplementary file 2 — 10.1186/s13613-016-0195-8 A Correlation between IAP and operating time. B. Correlation between IAP and perfusion time. C Correlation between IAP and aorta occlusion time. [file 13613_2016_195_MOESM2_ESM.pdf]
